# Dehydration Alters Transcript Levels in the Mosquito Midgut, Likely Facilitating Rapid Rehydration following a Bloodmeal

**DOI:** 10.3390/insects14030274

**Published:** 2023-03-09

**Authors:** Christopher J. Holmes, Elliott S. Brown, Dhriti Sharma, Matthew Warden, Atit Pathak, Blaine Payton, Quynh Nguyen, Austin Spangler, Jaishna Sivakumar, Jacob M. Hendershot, Joshua B. Benoit

**Affiliations:** Department of Biological Sciences, University of Cincinnati, Cincinnati, OH 45221, USA

**Keywords:** *Aedes aegypti*, aquaporin, bloodfeeding, ecdysteroid kinase, ion transport, osmolality, transcriptomics

## Abstract

**Simple Summary:**

Female mosquitoes have been using the blood of their hosts to produce eggs for millions of years. As humans have become much more abundant in recent millennia, many mosquito species have adapted to bloodfeeding on humans, especially in drier areas where rehydration sources may not be as abundant. Some species, such as the yellow fever mosquito, *Aedes aegypti*, have even developed a distinct preference for human hosts. Unfortunately for us, these mosquitoes are also known to spread several pathogens during bloodfeeding, resulting in occurrences of yellow fever, dengue, Zika, chikungunya, as well as other diseases. Although decades of research have focused on these mosquitoes, more research is needed to understand how these mosquitoes are processing bloodmeals in the low humidity conditions where they often reside. In this study we examined the midgut of *A. aegypti* mosquitoes to determine how bloodmeal utilization changes after exposure to low humidity conditions and ultimately found that these mosquitoes can quickly and efficiently rehydrate through bloodfeeding. These results indicate that *A. aegypti* can rely on human bloodmeals to rehydrate in low humidity conditions when other resources may be scarce, potentially resulting in altered disease transmission rates.

**Abstract:**

The mosquito midgut is an important site for bloodmeal regulation while also acting as a primary site for pathogen exposure within the mosquito. Recent studies show that exposure to dehydrating conditions alters mosquito bloodfeeding behaviors as well as post-feeding regulation, likely altering how pathogens interact with the mosquito. Unfortunately, few studies have explored the underlying dynamics between dehydration and bloodmeal utilization, and the overall impact on disease transmission dynamics remains veiled. In this study, we find that dehydration-based feeding in the yellow fever mosquito, *Aedes aegypti*, prompts alterations to midgut gene expression, as well as subsequent physiological factors involving water control and post-bloodfeeding (pbf) regulation. Altered expression of ion transporter genes and aquaporin 2 (AQP2) in the midgut of dehydrated mosquitoes as well as the rapid reequilibration of hemolymph osmolality after a bloodmeal indicate an ability to expedite fluid and ion processing. These alterations ultimately indicate that female *A. aegypti* employ mechanisms to ameliorate the detriments of dehydration by imbibing a bloodmeal, providing an effective avenue for rehydration. Continued research into bloodmeal utilization and the resulting effects on arthropod-borne transmission dynamics becomes increasingly important as drought prevalence is increased by climate change.

## 1. Introduction

Numerous studies over the last century have investigated the relationships between mosquitoes and relative humidity [1,2,3,4,5,6,7,8,9,10,11,12,13,14,15]. However, only a subset of those studies has investigated the physiological effects of low relative humidity on mosquito biology, with an even smaller subset controlling for and directly studying the impacts of relative humidity on mosquitoes. This disparity warrants further exploration, especially considering that weather conditions are a direct cause of dehydration in mosquitoes, and that incorporation of weather conditions into models may account for up to 80% of the weekly variation in mosquito infection [1,16].

Recent studies implicate dehydration stress in water and nutrient depletion, as well as in the compensatory mechanisms (e.g., increased bloodmeal retention) required to offset those detriments [1,7,17]. Unfortunately, these identified mechanisms have been predicted to alter disease propagation dynamics within the vector and through host-vector interactions [1,7]. For example, previous findings indicate that nutrient reserves in the northern house mosquito, *Culex pipiens*, decreased as dehydration exposure increased, resulting in reductions to mosquito survival and reproduction [8]. Conversely, fortified nutritional reserves have been shown to improve longevity and increase resistance to pathogen challenge [18]; but direct connections between dehydration and disease transmission dynamics remain unexplored. It is paramount to understand the specifics on how humidity drives alterations in mosquito physiology as well as the biological components and underlying compensatory mechanisms required to offset any related detriments.

Compensatory behaviors are well documented within mosquitoes, with an early study on *Anopheles* species showing that blood digestion increased during the hot season [15] and later studies demonstrating that a bloodmeal could be utilized for nutritional supplementation beyond egg production [19,20]. Hagan et al. (2018) began investigating the potential for compensatory mechanisms in dehydrated mosquitoes, finding that biting propensity and carbohydrate metabolism was altered in dehydrated *C. pipiens*, culminating in a predicted increase to West Nile virus (WNV) transmission [1]. Holmes et al. (2022) continued this line of research, finding in a recent study with *C. pipiens* and *A. aegypti* that dehydration prompted increases in bloodfeeding propensity and greater water content retention from a bloodmeal, resulting in improved survival for bloodfed mosquitoes in dehydrating conditions [7]. These responses to dehydration were predicted to increase compensatory bloodfeeding as a response to lost water, ultimately altering the vectorial capacity of both *C. pipiens* and *A. aegypti* [7]. 

When incorporated into models, disease transmission has been found to be strongly influenced, and predicted, by factors such as environmental stressors [21], viral transmission [22,23], differential expression of genes [24], and the interactions between those factors [1]. Considering the reliance of various disease transmission models on relative humidity as a factor, as well as the numerous implications of relative humidity on mosquito physiology and behavior [17], more research must be aimed at addressing the direct effects of water loss (i.e., dehydration) on mosquitoes. To continue addressing this lapse in research, our study incorporated transcriptomic analyses and physiological assays to address the biological effects of dehydration stress on early bloodmeal processing in *A. aegypti*. Specifically, this study developed transcriptomic profiles for the midguts of *A. aegypti* subjected to dehydration stress in relation to bloodfeeding, facilitating a better understanding of the compensatory mechanisms underlying physiological alterations. Understanding the interactions of a bloodmeal within the midgut of a dehydrated mosquito may offer insights into potential permissibility differences in the gut (e.g., through altered regulatory mechanisms), with possible implications for disease transmission dynamics. Regardless, understanding the effect that a natural stressor like dehydration has on the midgut further necessitates the inclusion of environmental effects in disease dynamics. This study used next-generation sequencing to determine underlying genes involved in pre- and post-dehydration bloodmeal regulation in *A. aegypti*. The results of this experiment revealed ion transporters, AQP2, RNA regulation, and kinase involvement in dehydration and bloodfeeding exposures within the midgut. These findings, in addition to those of stabilizing osmolality and unaltered midgut size or micronutrients, provide a more thorough understanding of the mechanisms that drive fluid acquisition and retention in dehydrated mosquitoes.

## 2. Materials and Methods

Mosquito husbandry: Mosquito larvae were reared according to standard practices on ground fish food (Tetramin) with added yeast extract (Fisher). Adult *A. aegypti* mosquitoes (Rockefeller strain) were reared under insectary conditions (27 °C, 80% RH; saturation vapor pressure deficit (SVPD) = 0.71 kPa) in 12 × 12 × 12” cages (BioQuip) with a 16 h:8 h light:dark cycle and unlimited access to DI water- and 10% sucrose solution-soaked cotton wicks *ad libitum*, unless otherwise stated. 

Relative humidity exposure protocol: Similar to Holmes et al., (2022), mosquitoes were subjected to desiccators containing controlled relative humidity conditions at 27 °C with 75% RH (dehydrating condition; SVPD = 0.89 kPa) or 100% RH (non-dehydrating condition; SVPD = 0.00 kPa) by being placed in groups of 50 into mesh-covered 50 mL centrifuge tubes. These humidity-controlled mosquitoes were held under desiccator conditions without access to water or sucrose solution for 18 h before being subjected to downstream procedures. A relatively high relative humidity of 75% in the dehydrated group was selected to represent slow, steady dehydration, while 100% RH was used for the non-dehydrating condition so that no water was passively lost to the environment.

Mosquito midgut processing for transcriptomic analyses: After RH treatment, mosquitoes were released into 12 × 12 × 12” cages (BioQuip) and permitted to bloodfeed to repletion (approximately 20 min) on a live human host (27-year-old male, leg; IRB, University of Cincinnati) or not permitted to bloodfeed but with a human leg just outside the cage. These conditions resulted in four different groups: N1, non-bloodfed/non-dehydrated (control) group; Y1, bloodfed/non-dehydrated group; N7, non-bloodfed/dehydrated group; Y7, bloodfed/dehydrated group. Three hours (±1 h) pbf, mosquitoes were dissected and the midguts from approximately 15 different mosquitoes were pooled and placed into Trizol (Invitrogen) held on ice. Digestion of blood occurs around 4 h pbf [25] and diuresis is well underway within 2 h [26,27], so dissections 3 h post-bloodmeal were chosen to encompass differentially expressed genes related to altered blood digestion/water retention. Pooled midguts were homogenized (Benchmark, BeadBlaster 24), in Trizol and stored at −70 °C until all samples were collected. RNA was extracted with Trizol according to manufacturer’s protocols and cleaned with a RNeasy Mini Kit (Qiagen). DNase (Ambion, Turbo-DNA-free) was used to remove genomic DNA, RNA concentration was determined with a Nanodrop 2000 (Fisher), cDNA libraries were generated (Illumina, TruSeq), and next-generation sequencing was conducted at the Cincinnati Children’s Hospital Medical Center’s DNA Sequencing and Genotyping Core. Samples can be found in the Sequence Read Archive (SRA) Database (BioProject ID: PRJNA851095).

Gene expression analyses: Samples were analyzed through three separate pipelines using recommended settings throughout: CLC Genomics Workbench 12.1 (CLC Bio, Boston, MA, USA), DESeq2-Kallisto, and DESeq2-Sailfish. All pipelines used the published *A. aegypti* RefSeq assembly (accession: GCF_002204515.2) as reference [28]. The latter two pipelines included importing samples into Galaxy [29], checking for quality with FastQC [30], trimming with Trimmomatic [31], and analyzing with Kallisto [32] or Sailfish [33], before utilization of DESeq2 [34]. Significantly expressed genes were determined by Benjamini-Hochberg procedure (*p*-value < 0.01), the genes identified by any pipeline are provided in (Supplementary Table S1), and the DESeq2 pipeline comparisons with mean normalized expression across all samples and log2 fold-changes are included in (Supplementary Table S2). Transcriptomic methods revealed sufficient coverage, with approximately 75–105 million paired-end reads per sample (Table 1). Gene ontology (GO) terms were generated by importing all significantly expressed genes (*p*-value < 0.01) with a log2 fold-change ≥ 1 identified by any pipeline (Supplementary Table S3) into g:Profiler [35]. Gene ontology terms were subsequently summarized with REVIGO [36] and visualized via CirGO [37] (Supplementary Table S4). Although all pipelines were used to identify genes for the GO analyses, only DESeq2 pipeline results were compared for downstream expressional analyses. The CLC pipeline protocol included calculated mean expression values of zero for numerous genes, resulting in comparative fold-changes of infinity. However, in the DESeq2 pipelines, genes with expression values of zero were not included as part of the analysis, reducing the false positive identification rate of differentially expressed genes. Due to our smaller sample sizes and these differences in pipeline methodology, only the more conservative DESeq2 pipelines were utilized for further analysis. To control for small sample sizes (i.e., two and three replicates per group), the DESeq2 package normalizes gene counts with a median-of-ratios scaling method to determine gene- and sample-specific factors that account for sequencing depth and library composition [34,38,39]. After DESeq2 used the median-of-ratios method to normalize gene expression across all groups, we primarily utilized the log2 fold-change for comparative purposes [34]. This method allowed us to represent genes with a variety of different baseline expression levels while focusing on the genes with the most substantial differentially expressed genes between groups. Furthermore, to verify pipeline output, all mean expressions were transformed with log2, regardless of group comparison, and were compared between the DESeq2-Kallisto and DESeq2-Sailfish pipelines. The pipeline outputs for both of these methods were found to be considerably correlated (n = 181, r = 0.921, *p*-value < 0.00001; Supplementary Figure S1).

Osmolality procedures: In addition to the two RH treatments, a post-dehydration exposure group was also analyzed 1 h after taking a bloodmeal. Bloodfeeding was completed by filling artificial (Hemotek) reservoirs with chicken blood (Pel-Freez Biologicals), covering with parafilm (Sigma-Aldrich), warming to 37 °C, introducing the covered reservoir to 12 × 12 × 12” cages (BioQuip) without access to water or sucrose solution for 1 h, and allowing the dehydrated mosquitoes to feed to repletion [40]. Before use, chicken blood was held at −20 °C and then permitted to thaw at 4 °C. One hour after conclusion of RH treatment or post-RH treatment blood feeding, mosquito hemolymph was extracted for osmolality measurement with a vapor pressure osmometer (Wescor Vapro 5600, EliTech).

Midgut volume quantification: Mosquitoes were subjected to non-dehydrating and dehydrating conditions as before with an additional group of mosquitoes from a colony enclosure with indiscriminate age and hydration status. These mosquitoes were bloodfed as before with an artificial feeder (Hemotek) filled with chicken blood (Pel-Freez Biologicals). Within 1 h pbf, mosquitoes were knocked out with CO_2_, dissected (N = 86) in phosphate-buffered saline (PBS), and photographed (Dino-Lite). Micrometer measurements were calibrated and determined in GIMP [41], before volume was approximated as an ellipsoid (4/3 × π × W^2^ × L).

Nutritional assays: Briefly, nutritional assays for lipid, glycogen, and trehalose levels were adapted from previous studies [42,43,44] and combined to allow for technical and biological replication. After relative humidity treatments, additional cohorts were permitted access to water and 10% sucrose solutions *ad libitum* for 24 h to represent recovery conditions from these treatments. The colony group in this context represents *A. aegypti* that were subjected to only colony conditions and not any additional RH treatment. For quantification, mosquitoes were collected from the same group, placed in a freezer until death (−20 °C), added in groups of 4 to STE buffer (2% Na_2_SO_4_), homogenized (Benchmark, BeadBlaster 24), and aliquoted for lipid (100 μL), trehalose (150 μL), and glycogen (150 μL). Six groups in biological triplicates and two standard curves in technical duplicate were distributed across two 96-well plates (Zinsser). Absorbance was determined on a microplate reader (Biotek, Synergy H1) at 525 and 625 nm for lipids and carbohydrates respectively. Due to the nested nature of the biological sample replicates, each group was replicated at least thrice on the two-plate design. 

Statistical analyses: Data management was completed in Excel [45] and R [46] through plyr [47], tidyr [48], dplyr [49], and Rmisc [50] packages. Figures were made in R using ggplot2 [51], in Excel [45], and with CirGO, before finalization in GIMP [41] and Inkscape [52]. Tables were made in Excel [45]. R (version 4.0.2) was used to complete appropriate statistical analyses [46].

## 3. Results

**Gene ontology reveals slight differences between midgut groups.** Our groups consisted of non-bloodfed (N), and bloodfed (Y) mosquitoes held at either 75% RH (7) or 100% RH (1). Our analyses identified hundreds of genes with differentially expressed transcripts between midgut group comparisons, revealing relatively constrained functionality within the midgut regardless of dehydration or bloodfeeding (Table 2). Despite the three-fold number of genes identified between the dehydrated and non-dehydrated midguts of non-bloodfed *A. aegypti* (237 genes), the comparison between dehydrated and non-dehydrated bloodfed midguts had the lowest number of differentially expressed genes, with less than 80 total genes identified (Table 2). These comparisons underscore the similarities in dehydrated and non-dehydrated midgut functionality within three hours pbf (Table 2).

**Figure 1:**
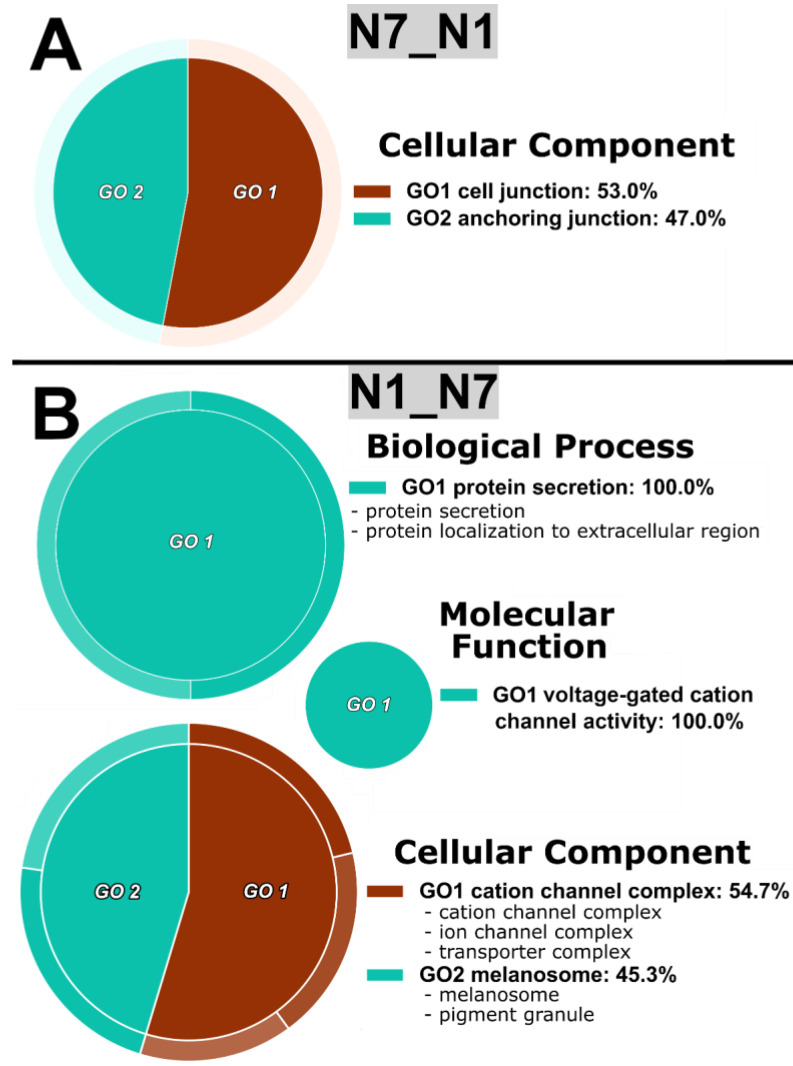
Functional enrichment analyses for non-bloodfed *A. aegypti* midguts. (**A**), circular gene ontology (CirGO) representations of reduced and visualized gene ontology (REVIGO) terms in the non-bloodfed/dehydrated group over the non-bloodfed/non-dehydrated group (N7_N1); (**B**), CirGO-REVIGO representations for the non-bloodfed/non-dehydrated group over the non-bloodfed/dehydrated group (N1_N7). REVIGO groupings are included in Supplementary Table S3 and significant g:Profiler terms are included in Supplementary Table S4 with “intersections” indicating the genes responsible for GO categorization. CLC labels represent significant transcripts identified with the QIAGEN CLC pipeline; DK, the DESeq2-Kallisto pipeline; and DS, the DESeq2-Sailfish pipeline.

All comparisons showed GO differences except for the contrasts between Y7 and Y1 groups, indicating that regardless of the level of dehydration status experienced in this study, bloodmeal processing in the midgut was remarkably similar (Figure 1; Supplementary Figure S2). The primary non-bloodfed N7_N1 comparison revealed cell and membrane interactions (Figure 1A), while the N1_N7 comparison showed persistent changes to ion channel activity (Figure 1B). The N1_Y1 comparison showed differences in developmental and regulatory genes (Supplementary Figure S2A), Y1_N1 revealed GO terms consistent with bloodmeal breakdown (Supplementary Figure S2B), N7_Y7 showed changes in protein binding and transcription (Supplementary Figure S2C), and Y7_N7 also uncovered GO terms associated with bloodfeeding as well as a number of terms relating to snRNPs and RNA functionality (Supplementary Figure S2D).

**Figure 2:**
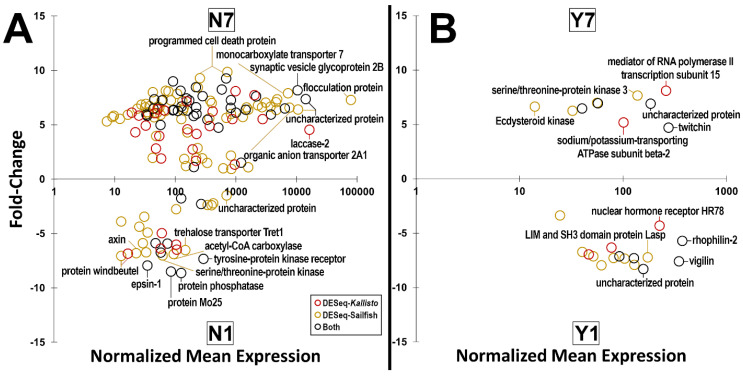
Log2 fold-change comparisons and median-of-ratios normalized mean expression averages across all samples for all differentially expressed genes identified by DESeq2 pipelines. (**A**), comparison between the non-bloodfed/dehydrated group over the non-bloodfed/non-dehydrated group (N7_N1); (**B**), comparison between the bloodfed/dehydrated group over the bloodfed/non-dehydrated group (Y7_Y1). Yellow circles denote genes that were identified through the DESeq-Sailfish pipeline; red circles, DESeq-Kallisto pipeline; and black circles were genes identified by both pipelines, with the highest mean expression pipeline used. Significantly expressed transcripts are included in Supplementary Table S1 and sample-specific normalized mean expression values are included in Supplementary Table S5.

In both the dehydrated and non-dehydrated comparisons between bloodfed and non-bloodfed *A. aegypti*, numerous transcripts directly associated with bloodmeal processing (e.g., trypsin, peritrophin, etc.) were upregulated in the bloodfed group, while a limited and lowly expressed set were significantly differentiated in the non-bloodfed group (Supplementary Figure S3). When comparing non-bloodfed groups, dehydrated *A. aegypti* had considerably more, and more highly expressed, transcripts than the non-dehydrated group (Figure 2A). In our dehydrated comparison (Supplementary Figure S3B), the non-bloodfed group also showed considerably higher expression than the non-bloodfed, non-dehydrated group in a similar comparison (Supplementary Figure S3A; Table S2). The dehydrated group also significantly expressed transcripts related to transporters and apoptosis while the non-dehydrated control had lowly-expressed phosphatases with high fold-changes (Figure 2A). When comparing bloodfed groups, there were only a couple dozen differentially expressed genes between the non-dehydrated and dehydrated groups, while all the transcripts had low mean expression values (Figure 2B). Furthermore, the non-dehydrated bloodfed group consisted of transcripts encoding cytoskeletal/structural elements (e.g., rhophilin-2, Lasp, etc.) and the dehydrated bloodfed group featured differential regulation of ion transporters, kinases, and an aquaporin (Figure 2B). The aquaporin gene AQPcic (AAEL003550-RA), also referred to as aquaporin 2 (AQP2) [53], was the only aquaporin differentially regulated in any of our groups, identified by the DESeq2-Kallisto pipeline to be down regulated in the Y7 group when compared to both the N1 and the N7 groups. Despite having lower expression than the non-bloodfed groups, the Y7 group had comparable expression to the Y1 group. This is consistent with previous findings that AQP2 is downregulated in the midgut post-bloodfeeding [26]. The dehydrated comparison between non-bloodfed and bloodfed *A. aegypti* showed stark similarities to the non-dehydrated bloodfeeding comparison in regard to bloodmeal processing (Supplementary Figure S3). 

**Figure 3:**
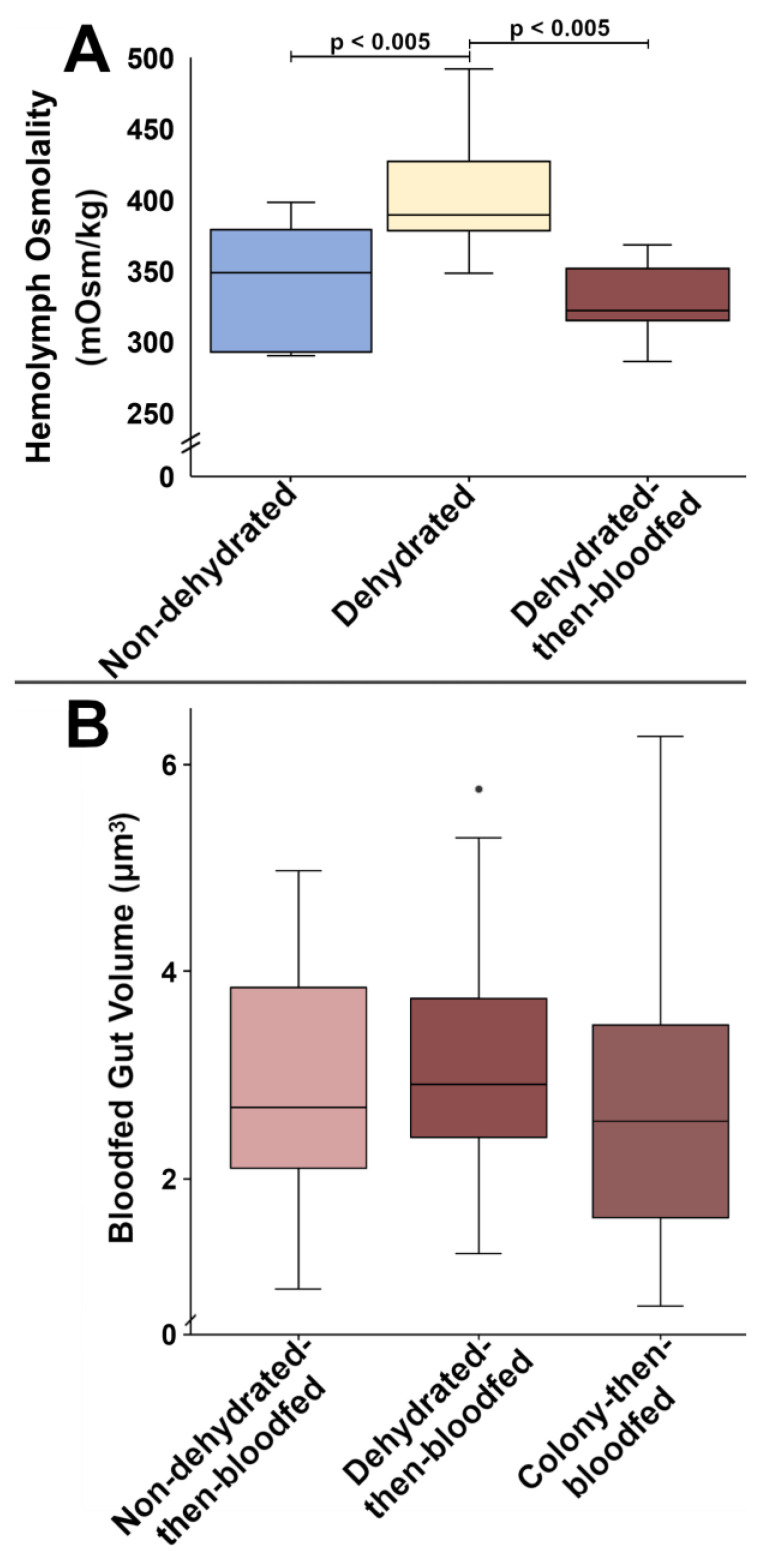
Hemolymph osmolality and bloodfed midgut volume for *A. aegypti* subjected to various treatments. (**A**), hemolymph osmolality for non-dehydrated (N1), dehydrated (N7), and post-dehydration bloodfed (Y7) *A. aegypti* (N = 30); (**B**), midgut size comparisons for bloodfed *A. aegypti* after 18 h of exposure to non-dehydrating (Y1), dehydrating (Y7), or colony conditions (N = 86). Significance was determined via ANOVA and Tukey’s HSD test.

**Post-dehydration bloodfeeding shifts hemolymph osmolality back to control levels.** Osmolality in the hemolymph increased as mosquitoes lost water in the dehydrated group (N7), but within 1 h pbf, hemolymph osmolality returned to non-dehydrated (N1) control levels in dehydrated-then-bloodfed (Y7) mosquitoes (Figure 3A). No alterations to lipid, glycogen, or the primary hemolymph carbohydrate, trehalose, were identified (Supplementary Figure S4). Finally, no distinguishable volume changes were identified in the dissected midguts of non-dehydrated-then-bloodfed (Y1), dehydrated-then-bloodfed (Y7), nor colony-then-bloodfed mosquitoes (Figure 3B).

## 4. Discussion

Through bloodfeeding, mosquitoes have been afforded the flexibility to regulate nutrients, reproductive output, survival, and more when compared to non-bloodfeeding organisms [54,55]. For example, female mosquitoes with diminished nutritional reserves are capable of diverting nutrients from a bloodmeal to supplement existing nutrient levels, but do so at the expense of reproductive output [56]. Likewise, stress related to teneral nutritional reserves may result in differentially utilized nutrients at a later time [25]. It is therefore understandable that mosquitoes stressed with acute or persistent dehydration have adapted numerous mechanisms to combat this influence [17]. A recent study investigating the physiological effects of dehydration demonstrated that water loss plays an integral role in mosquito reproduction, survival, water content regulation, and vectorial capacity [7]. In this study, we expand on these findings by exploring the potential underlying mechanisms by which these physiological changes may occur, through investigation of transcriptomic, volumetric, and osmolality changes at the midgut interface.

A previous study on the whole-body transcriptome of non-bloodfed dehydrated *C. pipiens* showed that many significantly upregulated pathways were related to carbohydrate metabolism [1]. These carbohydrate metabolism pathway alterations clearly corroborate the findings in another study showing that repeated bouts of dehydration resulted in reduced levels of stored carbohydrates and lipids in *C. pipiens* [8]. When sucrose solution and water were withheld and mosquitoes were permitted or prohibited to bloodfeed, proteins were consistently altered [7], but our research showed that other micronutrients including trehalose, glycogen, and lipids were no different between groups (Supplementary Figure S4). The lack of significant changes to nutrition were likely the result from the short interval in which the metabolic assays were completed (<18 h after experimental onset), but nonetheless represent responses to water loss, not nutritional depletion. When non-bloodfed *C. pipiens* were dehydrated to the point of 25% water loss (comparable water loss to our study) and were allowed to recover before nutrient levels were tested, there were likewise no differences in lipids, glycogen, protein, or sugar levels [8]. Although the midgut-specific focus of the sequencing in this research limited the breadth at which carbohydrate metabolism pathways could be discovered, the resolution at which the expressional analyses were performed allowed us to thoroughly investigate the effects of bloodfeeding and dehydration at the intersection of the midgut. Through analysis of the underlying mechanisms, we have facilitated a more thorough understanding on how mosquitoes respond to dehydration stress in the context of (1) water and nutrient utilization and (2) bloodmeal protein utilization. This mechanistic knowledge provides much needed context for recent discoveries involving the effects of dehydration stress on survival, reproduction, and vectorial capacity, within medically-important mosquitoes species [1,7].

To process a bloodmeal, which is composed of 80–87% water and approximately 90% protein in the remaining dry mass, mosquitoes must promptly and efficiently regulate these abundant resources [57,58]. To facilitate water regulation in mosquitoes, aquaporins (AQPs) are found throughout the alimentary canal. *A. aegypti* possess six AQPs that are used to maintain water homeostasis by providing integral functions of water balance, such as the transport of water [26,53,59,60,61]. Under normal conditions, approximately 40% of water, sodium (Na), and chloride (Cl) derived from a bloodmeal are reportedly excreted within the first two hours pbf [27]. However, as *A. aegypti* become dehydrated, pbf diuresis substantially decreases [7], likely resulting in increased urine retention by the Malpighian tubules. Previous findings showed that *A. aegypti* excrete less during dehydration [7] and that the knockdown of AQPs resulted in reduced excretion which improved survival in desiccating conditions [59]. Whole-body knockdown of the aquaporins primarily associated with water transport, AQPs 1, 4, and 5, resulted in reduced excretion, knockdown of AQP2 did not [26]. Therefore, in a subsequent publication, desiccation tolerance was observed to be improved in *A. aegypti* with knockdown on AQPs 1, 4, and 5, while AQP2 was not included in this analysis [59]. However, the authors also note high expression of AQP2 in the midgut as well as a downregulation in the gene post-bloodfeeding [26,27,59]. This indicates that AQP2 does maintain some function involved in water transport, such as the dehydration of a bloodmeal, a part of the process where *A. aegypti* can reduce bloodmeal volume significantly (up to 75%) within a few hours [26,27,59]. Furthermore, an orthologous gene was found in *Anopheles gambiae*, AgAQP1 splice variant B, that likewise functions as a water channel and improved desiccation tolerance when expression was reduced [60,61]. In fact, most diuresis is completed within two hours post-bloodfeeding [27], and the differential expression of AQPs in the alimentary canal may be used to augment this process [59]. However, despite investigating AQP gene expression at 3 h post-bloodfeeding, the same authors found that AQPs were generally down-regulated 12-to-24 h post-bloodfeeding, and that the only AQP to be down regulated in the midgut 3 h post-bloodfeeding was AQP4 [26]. This indicates that the combination of dehydration and bloodfeeding likely prompted not only the expedited down regulation of AQP2 in the midguts of our dehydrated-then-bloodfed *A. aegypti*, but also that AQP4 may not have been as quickly down regulated. Interestingly, AQP4 directly contributed to diuresis regulation in *A. aegypti* while AQP2 did not [26,59], indicating that AQP2 may be more directly involved in the regulation of water out of the gut, while regulation of AQP4 may be more integral in water processing in the Malpighian tubules. More studies investigating the influences of AQPs on bloodfeeding regulation and desiccation tolerance are needed.

This information coupled with our osmolality findings taken one-hour pbf indicate that *A. aegypti* can exchange ions and extract water from a bloodmeal when necessary to combat dehydration. While ions are actively transferred through the midgut, as indicated by differential expression of ion transporters in this study, water transfer from the more dilute human blood into the hemolymph may occur passively due to osmolality differences in addition to facilitation by AQP2 [27,62]. The excessive quantities of protein and water in a bloodmeal afford flexibility to mosquitoes [54,55], and in desiccating conditions, excess water likely allows for rapid replacement of previously lost water. The increased retention of bloodmeal components, as seen in this study through reequilibrated hemolymph osmolality and transcriptional regulation of ion transporters, is also corroborated by previous studies reporting reduced diuresis as well as by high variability observed in the dry masses of dehydrated mosquitoes [this study,1,7]. Specifically, our study shows that numerous genes consistent with ion channel activity were differentially regulated between our non-dehydrated and dehydrated groups and that bloodmeal processing (e.g., trypsin, peritrophin) genes were differentially regulated in our bloodfed groups. Our osmolality data paired with the expression of ion transporters during *A. aegypti* dehydration, further underscores the importance of water content regulation in mosquitoes.

As for protein utilization, a considerable amount of enzymatic/proteolytic activity occurs in the ectoperitrophic space, and very little activity in the blood-filled midgut homogenates [63,64]. A number of these processes are implicated in our transcriptional analyses (e.g., peritrophin, trypsin, etc.). Additional transcripts such as AQP2, ion transporters, and kinases offer insight into the potential means through which *A. aegypti* may compensate for dehydration and bloodfeeding stress at the midgut interface. In our comparison between bloodfed groups, the dehydrated group had increased expression in a number of kinases over the non-dehydrated group. Of particular interest, one specific gene (AAEL012685-RC) encoded an ecdysteroid kinase (the family including ecdysteroid 22-kinase), which closely identifies with juvenile hormone-inducible proteins and hypothetical proteins found across an array of other medically-important mosquito species (e.g., *Anopheles gambiae*, *Culex pipiens*, *Aedes albopictus*, etc.; Supplementary Table S6). This may offer additional insight into the reduced egg production observed in dehydrated mosquitoes [7], or potentially into the veiled 20-hydroxyecdysone (20E) signaling pathway. Another over-expressed gene of interest identified in our Y1_Y7 comparison, vigilin (AAEL001421-RA), has been implicated in the formation of RACK1, which is involved in viral RNA binding for DENV genome amplification [65]. Considering the abundance of RNA-involved processes in our Y7_N7 comparison, especially regarding our Y1_N1 comparison, possibilities exist for interactions between imbibed pathogens and the genes expressed within dehydrated mosquitoes. However, more research is needed to address the potential for altered processing of a post-dehydration bloodmeal in the event that an imbibed bloodmeal were to contain pathogens such as Mayaro, Zika, or Dengue (DENV) viruses. 

Mosquitoes that underwent dehydration stress were predicted to increase WNV infections as a result of increased biting propensity, while in a similar finding, mosquitoes with reduced nutritional reserves had an increased propensity to orally transmit WNV infection [1,18]. We originally postulated that mosquitoes may compensate for dehydration stress by over-indulging on a bloodmeal, resulting in increased permissibility for imbibed pathogens via induced microperforations [66], but our volumetric analyses determined that the midgut was not overfilled immediately after bloodfeeding. These results indicate that the reduced diuresis *A. aegypti* produce when dehydrated [7] could not be explained by excess fluid in the midgut, and that hemolymph reequilibration is more likely. These findings, however, do not exclude the influences of gene regulation on pathogen interactions. It is possible that dehydration may prompt the supplementation of pbf water and nutritional reserves at the expense of reproduction [7,56], and that dehydration may also promote increased instances of refeeding in dehydrated mosquitoes, furthering the potential for additional pathogen exposure for both hosts and vectors. Similar to Armstrong et al. [66], these dehydration-prompted refeedings may promote microperforations to the midgut, resulting in increased pathogen dissemination. Pathogen dissemination may also be encouraged in dehydrated mosquitoes by expedited passage of bloodmeal components through the midgut barrier via active means such as transporter-facilitated efflux and/or via passive means down a concentration gradient with water from relatively dilute blood to the more concentrated hemolymph. Furthermore, the previously reported reduction to pbf diuresis in dehydrated mosquitoes may continue to alter pathogen interactions within the mosquito via increased bloodmeal retention [7]. To address these possibilities, more research should be completed on the direct influence of dehydration as well as the effects of dehydration-induced refeeding on midgut permissibility to, and downstream retention of, pathogens. Hopefully, these results may be used to continue addressing the gaps in knowledge regarding the impact of dehydration on arthropod-borne disease transmission that still exist. Additional information on the direct interaction between pathogens and dehydrated mosquitoes, especially at the midgut interface, is sorely needed.

## 5. Conclusions

Mosquitoes must meticulously regulate water content to maintain homeostasis, especially after imbibing a bloodmeal. These dynamics become particularly interesting in dehydrating conditions, with a recent study reporting that 70–90% of the largest bloodmeals taken by *A. aegypti* and *C. pipiens* (as indicated by hemoglobin content) were found in dehydrated mosquitoes [7]. However, in this study, we saw no indication of enlargement in dehydrated *A. aegypti* midguts, further indicating the expedited processing of post-dehydration bloodmeals. Taken together with the knowledge that *A. aegypti* are also known to reduce pbf diuresis when dehydrated [7], these results indicate an ability to begin bloodmeal processing for rehydration during or immediately after feeding. Specifically, AQP2 may be involved in the mitigation of water passage from a bloodmeal to be excreted when that water could be used for hydration purposes instead. *A. aegypti* may maintain water balance by altering AQP2 expression, in concert with other genes of interest, to selectively dispatch water from a bloodmeal for excretion, depending on hydration status. Therefore, we propose that while AQPs 1, 4, and 5 may be more directly involved in diuresis, AQP2 may assist in transporting the water from a bloodmeal for availability downstream in the alimentary canal. Taken together, these alterations may result in an overall greater intake and retention of a post-dehydration bloodmeal, all while lost water is replenished and maximum midgut size remains unsurpassed. Although *A. aegypti* did not undergo diuresis while feeding as *Anopheles* species do, alterations in GO pathways, underlying genes, bloodmeal processing, and retention in dehydrated *A. aegypti* indicate that similar processes may be involved. Considering the possibility of dehydrated mosquitoes to imbibe and expeditiously process pathogens alongside bloodmeal components, as well as the potential for more direct vector-pathogen interactions, more research on pathogen ingestion and dissemination in this context remains intriguing.

## Figures and Tables

**Table 1:** Descriptive information regarding sample composition and read counts of experimental groups. Sample numbers are provided in the respective column.

Group	Dehydration	Bloodfed	Sample	Paired-End Reads
N1	No	No	N1-2	75,496,800
			N1-3	88,761,156
Y1	No	Yes	Y1-1	89,322,470
			Y1-2	81,691,584
			Y1-3	74,120,060
N7	Yes	No	N7-1	105,818,594
			N7-2	105,385,942
			N7-3	82,647,520
Y7	Yes	Yes	Y7-1	95,531,032
			Y7-2	85,314,086

**Table 2:** Group comparison information regarding significantly expressed genes, Gene Ontology (GO) pathways, and REVIGO terms. Gene lists, GO pathways, and REVIGO terms were generated from transcripts identified by any pipeline. Specific information can be found in Supplementary Tables S1–S4. Group N1Y1 represents comparisons between the non-bloodfed/non-dehydrated and the bloodfed/non-dehydrated groups; N7N1, non-bloodfed/dehydrated and non-bloodfed/non-dehydrated groups; Y7Y1, bloodfed/dehydrated and bloodfed/non-dehydrated groups; N7Y7, non-bloodfed/dehydrated and bloodfed/dehydrated groups.

Group	Comparison	Genes	GO Pathways	REVIGO Terms
Y1N1	Y1/N1	145	7	4
	N1/Y1	62	3	3
N7N1	N7/N1	146	4	2
	N1/N7	91	13	5
Y7Y1	Y7/Y1	37	0	0
	Y1/Y7	40	0	0
N7Y7	N7/Y7	390	8	5
	Y7/N7	281	29	4

## Data Availability

Data for the transcriptomic samples can be found in the Sequence Read Archive (SRA) Database (BioProject ID: PRJNA851095).

## Supplementary Materials

The following supporting information can be downloaded at: https://www.mdpi.com/article/10.3390/insects14030274/s1, Figure S1: DESeq2 pipeline comparisons show considerable correlation in shared transcript expression; Figure S2: Functional enrichment analyses; Figure S3: Log2 fold-change and normalized mean expression comparisons for all significantly expressed genes identified by DESeq2 pipelines; Figure S4: Macronutrient levels for A. aegypti subjected to various treatments; Table S1: All significant comparisons (*p*-value < 0.01, log2 fold-change > 1) between groups; Table S2: All significant DESeq2-identified transcript comparisons (*p*-value < 0.01) between groups; Table S3: All significant REVIGO representatives for GO terms between groups; Table S4: All significant GO terms and KEGG pathways identified by g:Profiler between groups; Table S5: Normalized gene expression generated by the DESeq2-Sailfish and -Kallisto pipelines for individual samples, including average normalized expression; Table S6: BLAST information of proteins associated with an identified ecdysteroid kinase protein (gene ID AAEL012685-RC).
