# Supplementary material for: Dehydration Alters Transcript Levels in the Mosquito Midgut, Likely Facilitating Rapid Rehydration following a Bloodmeal
Source: Insects. 2023 Mar 9;14(3):274. doi: 10.3390/insects14030274 (PMC10056721; doi:10.3390/insects14030274)
Supplement: Supplementary file 1 [file insects-14-00274-s001.zip › Supplemental_Figures.pdf]

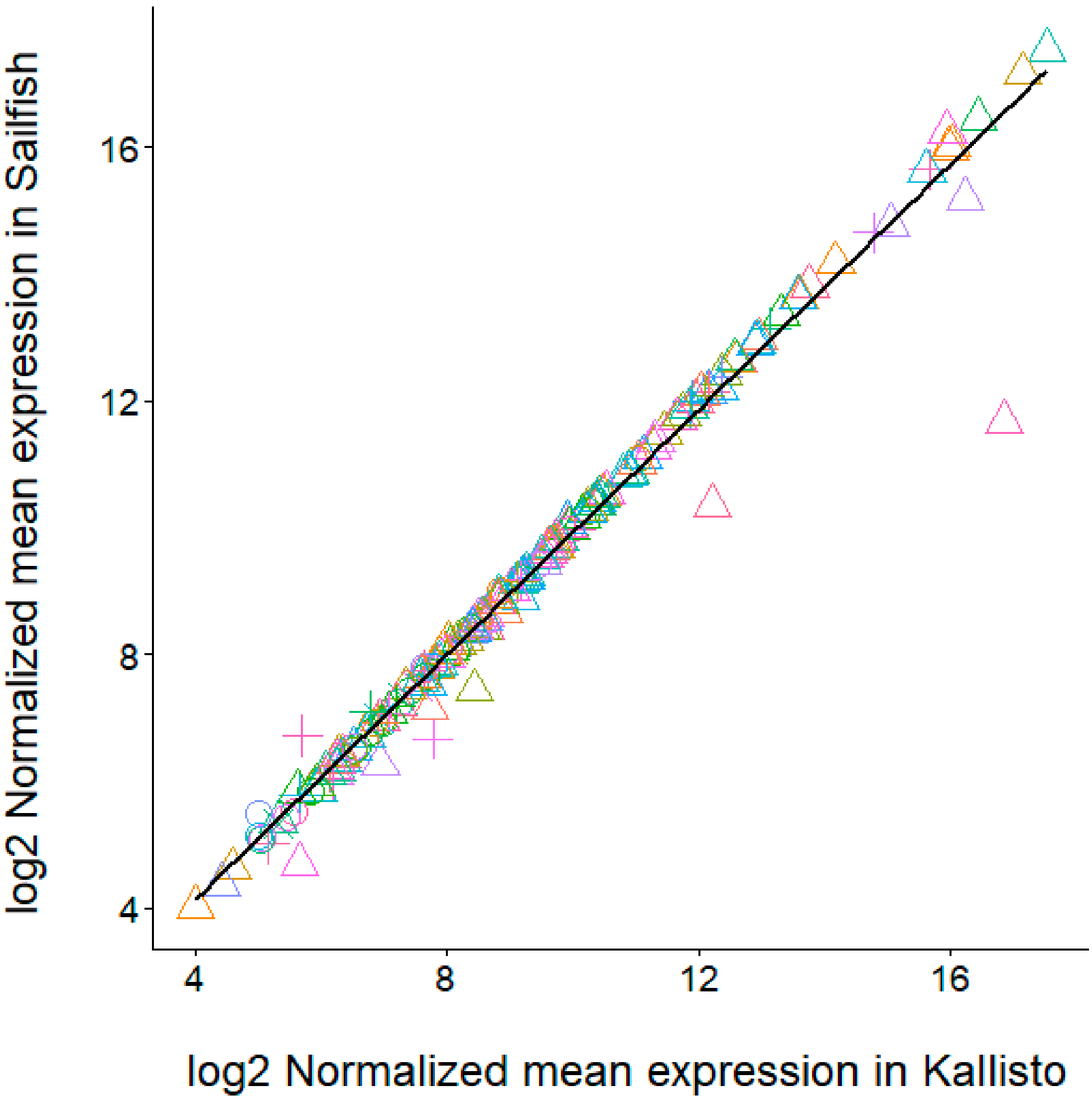

Supplementary Figure S1: DESeq2 pipeline comparisons show considerable correlation in shared transcript expression. The Y1_N1 comparison is denoted by circles; Y1_Y7, triangles; N7_N1, squares; N7_Y7, crosses.

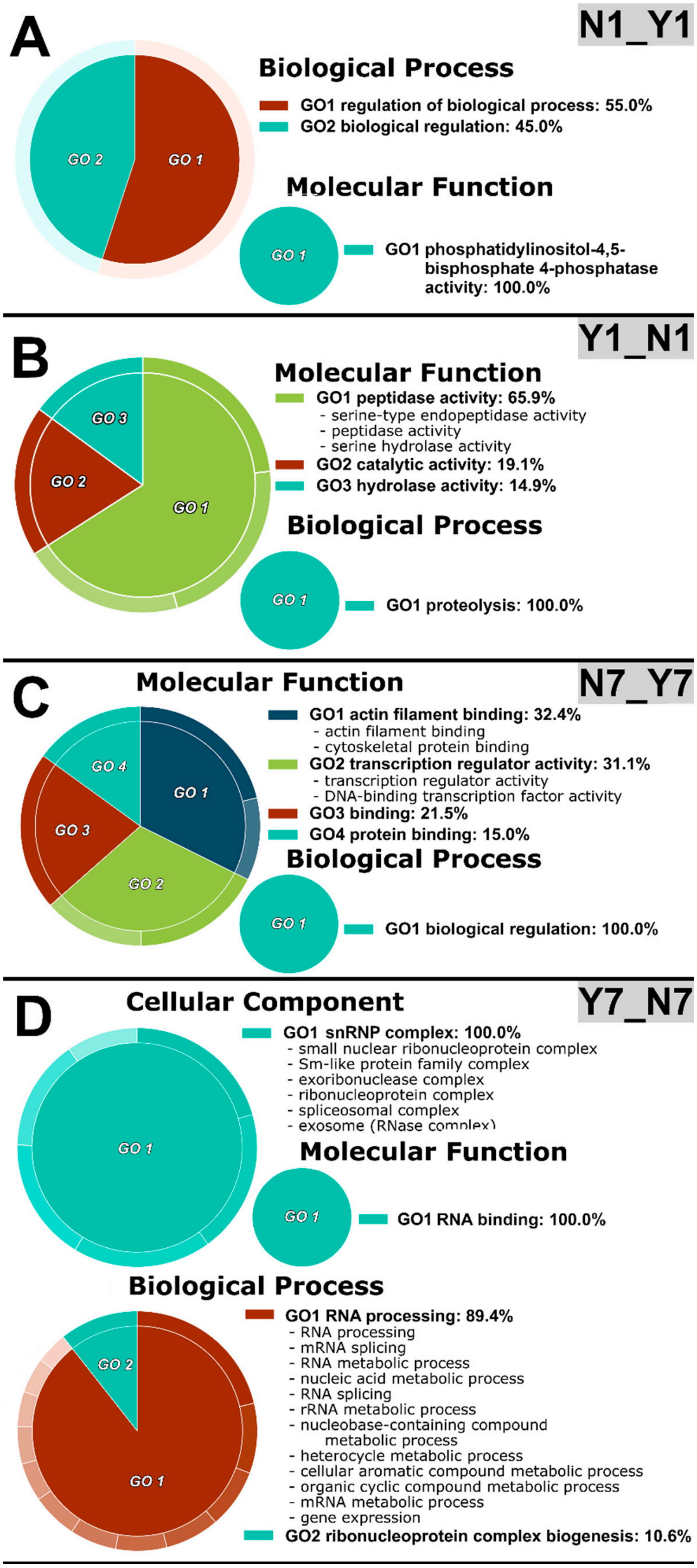
A
N1_Y1
Biological Process
GO 1
GO 2
GO1 regulation of biological process: 55.0%
GO2 biological regulation: 45.0%
Molecular Function
GO 1
GO1 phosphatidylinositol-4,5-bisphosphate 4-phosphatase activity: 100.0%
B
Y1_N1
Molecular Function
GO 1
GO 2
GO 3
GO1 peptidase activity: 65.9%
- serine-type endopeptidase activity
- peptidase activity
- serine hydrolase activity
GO2 catalytic activity: 19.1%
GO3 hydrolase activity: 14.9%
Biological Process
GO 1
GO1 proteolysis: 100.0%
C
N7_Y7
Molecular Function
GO 1
GO 2
GO 3
GO 4
GO1 actin filament binding: 32.4%
- actin filament binding
- cytoskeletal protein binding
GO2 transcription regulator activity: 31.1%
- transcription regulator activity
- DNA-binding transcription factor activity
GO3 binding: 21.5%
GO4 protein binding: 15.0%
Biological Process
GO 1
GO1 biological regulation: 100.0%
D
Y7_N7
Cellular Component
GO 1
GO1 snRNP complex: 100.0%
- small nuclear ribonucleoprotein complex
- Sm-like protein family complex
- exoribonuclease complex
- ribonucleoprotein complex
- spliceosomal complex
- exosome (RNase complex)
Molecular Function
GO 1
GO1 RNA binding: 100.0%
Biological Process
GO 1
GO 2
GO1 RNA processing: 89.4%
- RNA processing
- mRNA splicing
- RNA metabolic process
- nucleic acid metabolic process
- RNA splicing
- rRNA metabolic process
- nucleobase-containing compound metabolic process
- heterocycle metabolic process
- cellular aromatic compound metabolic process
- organic cyclic compound metabolic process
- mRNA metabolic process
- gene expression
GO2 ribonucleoprotein complex biogenesis: 10.6%

Supplementary Figure S2: Functional enrichment analyses. A, CirGO-REVIGO representations for the non-bloodfed/non-dehydrated group over the bloodfed/non-dehydrated group (N1_Y1); B, CirGO-REVIGO representations for the bloodfed/non-dehydrated group over the non-bloodfed/non-dehydrated group (Y1_N1); C, CirGO-REVIGO representations for the non-bloodfed/dehydrated group over the bloodfed/dehydrated group (N7_Y7); D, CirGO-REVIGO representations for the bloodfed/dehydrated group over the non-bloodfed/dehydrated group (Y7_N7). REVIGO groupings are included in Supplementary Table 3 and significant g:Profiler terms are included in Supplementary Table 4 with “intersections” indicating the genes responsible for GO categorization. CLC labels represent significant transcripts identified with the QIAGEN CLC pipeline; DK, the DESeq2-Kallisto pipeline; and DS, the DESeq2-Sailfish pipeline.

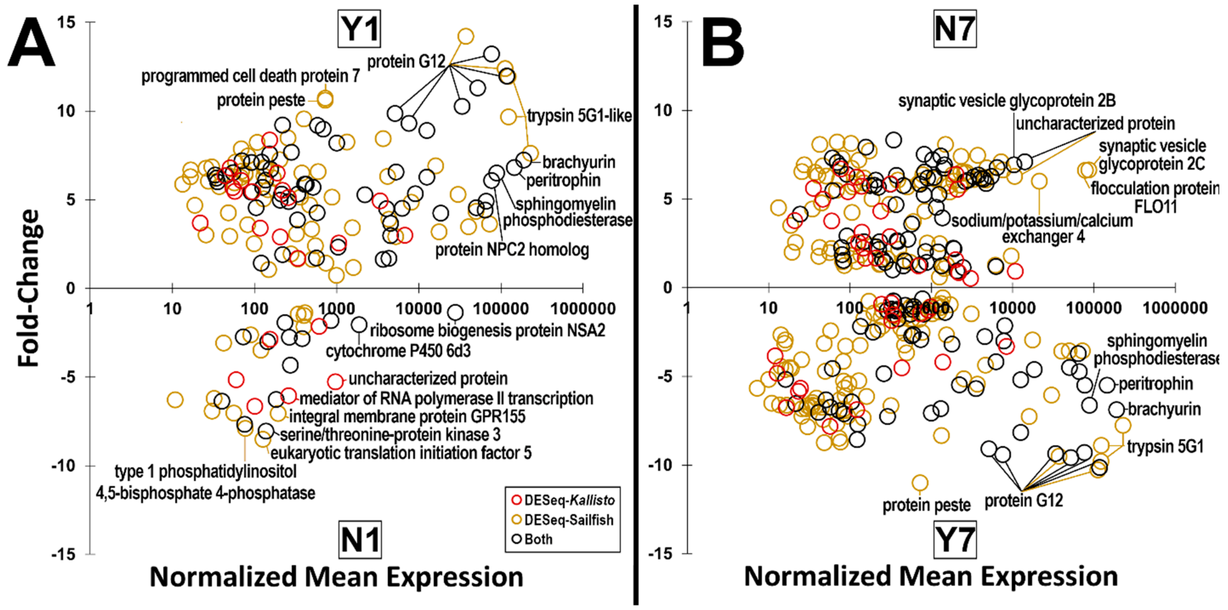

<u>**Supplementary Figure S3:**</u> Log2 fold-change comparisons and median-of-ratios normalized mean expression averages across all samples for all differentially expressed genes identified <u>by DESeq2 pipelines. A, comparison between the bloodfed/non-dehydrated group over the non-bloodfed/non-dehydrated group (Y1_N1); B, comparison between the non-bloodfed/dehydrated group over the bloodfed/dehydrated group (N7_Y7). Yellow circles denote genes that were identified through the DESeq-Sailfish pipeline; red circles, DESeq-*Kallisto* pipeline; and black circles were genes identified by both pipelines, with the highest mean expression pipeline used. Significantly expressed transcripts are included in Supplementary Table 1</u> and sample-specific normalized mean expression values are included in Supplementary Table 5.

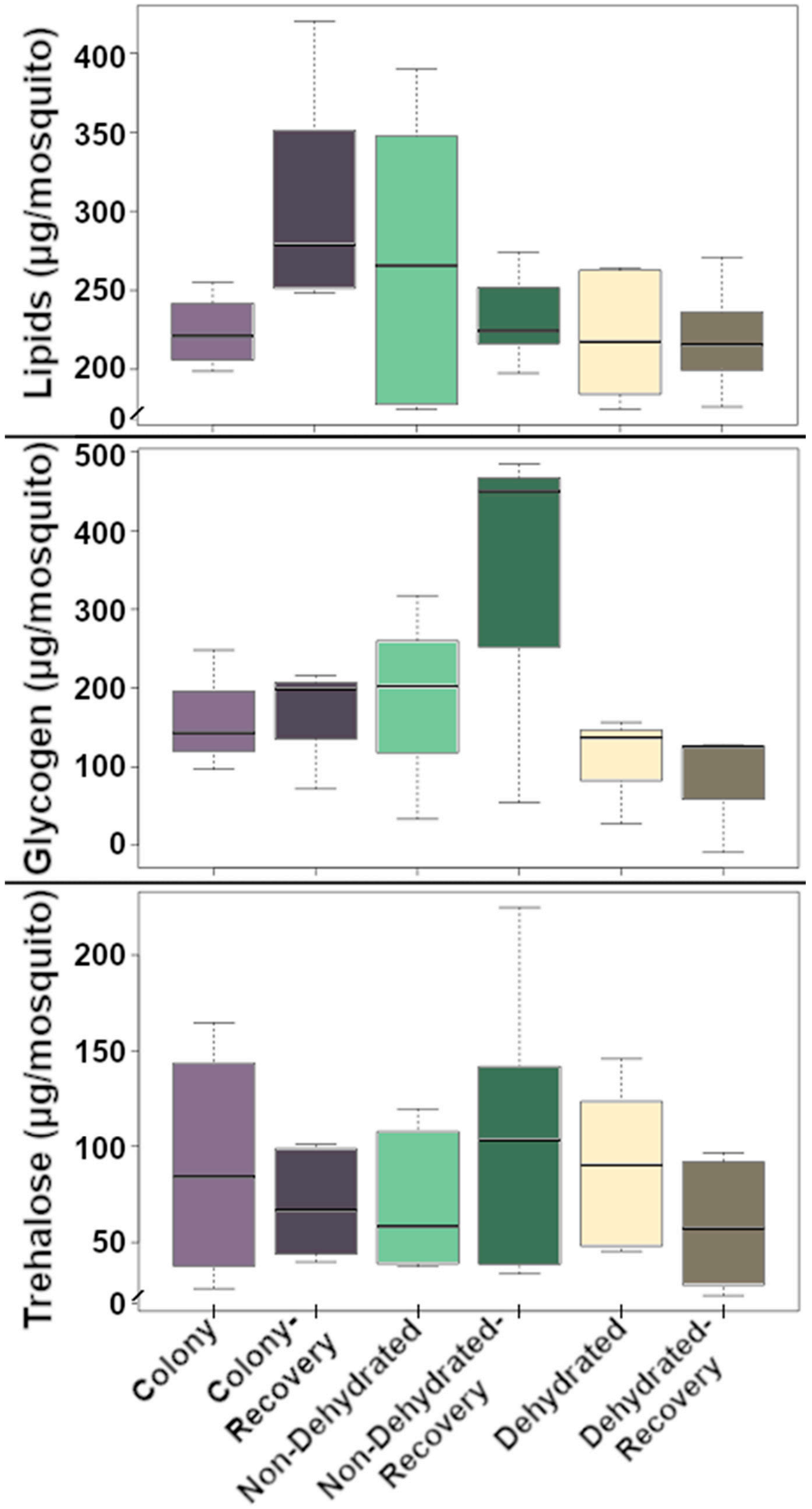

Supplementary Figure S4: Macronutrient levels for *A. aegypti* subjected to various treatments.

Supplementary Table S1: All significant comparisons (p-value < 0.01, log2 fold-change >1) between groups. Including gene transcripts, nucleotide ID, protein IDs, and relevant protein description. In each Excel worksheet, the initial term (e.g., Y1) represents increased log2 fold-change over the second term (e.g., N1) in a sequence (e.g., Y1_N1 represents Y1 gene expression over N1 expression). CLC, abbreviation for genes identified by the CLC pipeline; DK, DESeq2-Kallisto; DS, DESeq2-Sailfish; FC, log2 fold-change; -p, p-value.

Supplementary Table S2: All significant DESeq2-identified transcript comparisons (p-value < 0.01) between groups. Including gene transcripts, highest normalized mean expression across all samples, log2 fold-change, protein ID, and relevant protein description. Each Excel worksheet represents a group comparison. Red fill represents genes identified by the DESeq2-Kallisto pipeline; yellow, DESeq2-Sailfish; black, both DESeq2-pipelines.

Supplementary Table S3: All significant REVIGO representatives for GO terms between groups. Including GO term IDs, GO ID names, and REVIGO-assigned representative category. In each Excel worksheet, the initial term (e.g., Y1) represents increased expression over the second term (e.g., N1) in a sequence (e.g., Y1N1 represents Y1 gene expression over N1 expression), the following term (e.g., BP) denotes the GO abbreviation. GO, abbreviation for gene ontology; BP, biological process; CC, cellular component; MF, molecular function.

Supplementary Table S4: All significant GO terms and KEGG pathways identified by g:Profiler between groups. Including term names, term IDs, p-value, number of transcript intersections, and identified intersecting gene transcripts. In each Excel worksheet, the initial term (e.g., Y1) represents increased expression over the second term (e.g., N1) in a sequence (e.g., Y1N1 represents Y1 gene expression over N1 expression), the following term (e.g., BP) denotes the GO abbreviation. GO, abbreviation for gene ontology; BP, biological process; CC, cellular component; MF, molecular function; KEGG, Kyoto Encyclopedia of Genes and Genomes pathways.

Supplementary Table S5: Normalized gene expression generated by the DESeq2-Sailfish and -Kallisto pipelines for individual samples, including average normalized expression.

Supplementary Table S6: BLAST information of proteins associated with an identified ecdysteroid kinase protein (gene ID AAEL012685-RC).
